# Providing Flaxseed Oil but Not Menhaden Oil Protects against OVX Induced Bone Loss in the Mandible of Sprague-Dawley Rats

**DOI:** 10.3390/nu8100597

**Published:** 2016-09-24

**Authors:** Amanda B. Longo, Wendy E. Ward

**Affiliations:** Faculty of Applied Health Sciences, Brock University, St. Catharines, ON L2S 3A1, Canada; ll12xx@brocku.ca

**Keywords:** alveolar bone, mandible, micro-computed tomography, omega-3 fatty aids, alpha-linolenic acid, eicosapentaenoic acid, docosahexaenoic acid, ovariectomy, flaxseed oil, fish oil

## Abstract

Higher intakes of polyunsaturated fatty acids (PUFA) are associated with benefits at several skeletal sites in postmenopausal women and in rodent models, but the effect of PUFA-containing oils on tooth-supporting alveolar bone of the mandible has not been studied. Moreover, direct comparison of the effect of flaxseed oil (a source of alpha-linolenic acid (ALA)) and menhaden oil (a source of eicosapentaenoic acid (EPA) and docosahexaenoic acid (DHA)) is unknown. One-month old female Sprague-Dawley rats (*n* = 48) were randomized to and fed a diet containing flaxseed oil or menhaden oil from one to six months of age. At three months of age, rats were randomized to receive SHAM or ovariectomy (OVX) surgery (*n* = 12/diet). The inter-radicular septum below the first molar of the mandible was imaged at 6 months of age (study endpoint) using micro-computed tomography (μCT) at a resolution of 9 μm. As expected, OVX significantly reduced percent bone volume (BV/TV), connectivity density (Conn. D.), trabecular number (Tb. N.), and increased trabecular separation (Tb. Sp.) compared to SHAM rats (*p* < 0.001). However, post hoc analysis revealed these differences were present in rats fed menhaden oil but not those fed flaxseed oil. These results suggest that providing flaxseed oil, possibly through its high ALA content, provides protection against the OVX-induced alveolar bone loss in rats.

## 1. Introduction

The characteristic decline in bone mineral density (BMD) at the hip, spine, and wrist that is associated with an increased risk of fragility fracture, is also associated with tooth loss in postmenopausal women [1,2,3,4,5,6,7,8,9,10,11,12]. Having a higher number of teeth and functional dentition (i.e., having pairs of occluding teeth) is positively associated with aspects of a healthful diet and a higher Healthful Eating Index score in older adults [13,14,15,16,17]. Moreover, consuming a healthful diet has been linked to a reduced risk of many chronic diseases including osteoporosis [18,19,20,21]. Together, these findings suggest a cyclical relationship among a healthful diet, tooth loss, and osteoporosis.

Several large observational studies have highlighted positive associations between higher dietary intake of *n*-3 polyunsaturated fatty acid (PUFA) and a decreased risk of fracture risk [22,23] or an increase in total body, femur, or lumbar spine BMD [24,25,26,27] in postmenopausal women. These associations may be due to the anti-inflammatory properties of the *n*-3 PUFA [28] although exact mechanisms have not been determined. *N*-3 PUFA can exist in the diet either as the essential fatty acid, alpha-linolenic acid (ALA), or the longer chain eicosapentaenoic acid (EPA) and docosahexaenoic acid (DHA). ALA is commonly found in plant-based foods, such as flaxseed, walnuts, soy, and *n*-3 fortified foods. EPA and DHA can be converted in the body through relatively inefficient processes from ALA, or can be consumed in all types of fish and fish oils. In Canada and the US, the adequate intake of ALA for women over 50 years is 1.1 g/day and although there is no dietary reference intake for the long chain *n*-3 PUFA, it is recommended that up to 10% of the total fatty acid intake be consumed as EPA and DHA [29]. While total *n*-3 intake has been shown to benefit bone health in postmenopausal women, the relative contributions of different *n*-3 fatty acids, such as ALA or its long chain derivatives, EPA and DHA, have not been consistently demonstrated in the literature [28].

In humans, few studies have evaluated the impact of total dietary *n*-3 PUFA and oral health, but associations have been observed between higher intakes of EPA, DHA and fish oil and lower incidence of periodontal disease and tooth loss, and with greater healing after periodontal surgery [30,31,32,33,34]. Similar associations with ALA were either not investigated [31,32,33] or not observed [30,34]. Controlled trials investigating fish oil supplementation and oral health in adults are limited and have provided no definitive conclusions [35,36]. In addition, no randomized controlled trials (RCT) have investigated the effect of either flaxseed oil or ALA supplementation on oral health. The few RCTs that have investigated the relationship between flaxseed oil and bone health, either through measurement of biochemical markers of bone health or BMD at hip or spine in postmenopausal women, have not shown a benefit, possibly due to the relatively healthy state of the women studied [37,38,39]. RCTs with fish oil supplementation have resulted in either a beneficial change to markers of general bone health [40,41] or no difference compared to controls [42,43].

Ovariectomy (OVX) in the rat results in systemic bone loss and deterioration of the bone microarchitecture, similar to the loss of bone mass and structure in women following menopause [44]. Therefore, the OVX rat model is the approved preclinical model for the study of postmenopausal osteoporosis and its potential interventions, including nutritional strategies [45]. Although the etiology of tooth loss is multifactorial, hormone-induced bone loss of the alveolar bone supporting the teeth is a potential influencing factor [46]. Several studies have investigated the impact of OVX on the alveolar bone at multiple sites of the mandible, but only few have used micro-computed tomography (μCT), the gold standard for the quantification of bone microarchitectural changes [47,48,49,50,51,52,53]. These studies have consistently shown the characteristic decline in trabecular bone microarchitecture of the alveolar bone of the mandible compared to ovary-intact (SHAM) rats [47,48,49,50,51,52,53].

Therefore, the objective of this study was to determine whether flaxseed oil or menhaden oil would preserve the trabecular microarchitecture, a major determinant of the structural integrity of the alveolar bone, using μCT in the OVX rat model of postmenopausal osteoporosis. The dietary intervention was started prior to SHAM or OVX surgery, during a period of rapid growth, to mimic a lifelong exposure to the *n*-3 containing oils.

## 2. Materials and Methods

### 2.1. Animals and Diets

Twenty one-day old, female Sprague-Dawley rats (*n* = 48) were received from Charles River Canada (Sherbrooke, QC, Canada). Following one-week acclimatization, rats were randomized to receive a modified AIN93G diet containing 18% kcal from protein, 57% kcal from carbohydrate and 25% kcal from fat. The two intervention diets provided 11% fat by weight with an *n*-6 to *n*-3 PUFA ratio of 5:1 with linoleic acid (LA) from safflower oil as the *n*-6 source and either ALA from flaxseed oil (TD. 140813, Harlan Teklad Madison, WI, USA) or EPA and DHA from menhaden oil (TD. 140814, Harlan Teklad, Madison, WI, USA) as the *n*-3 source (*n* = 24/diet). The intervention diets were modified to mimic a physiologically relevant diet pattern that can be easily consumed as part of a healthful diet in humans. Diets were matched for total SFA, MUFA, and PUFA and were isocaloric (4.0 kcal/kg) (Table 1). At 12-weeks of age, rats were randomized within each diet (flaxseed oil or menhaden oil) to receive SHAM-control (*n* = 12/diet) or OVX (*n* = 12/diet) surgery. All rats were given *ad libitum* access to food and water and were doubly housed with rats of similar surgery type and diet, in ventilated cages in a controlled environment (12-h light and 12-h dark cycle; 20 °C). To avoid oxidation of dietary PUFA, all diets were packaged in vacuum-sealed bags at −20 °C until use, and diets were changed three times per week. Body weight was measured at six months of age, coinciding with study end point and food intake was measured over the final week of the study period. At six months of age, rats were sacrificed and the mandible was excised, cleaned of all soft tissue, wrapped in saline soaked gauze and stored at −80 °C until μCT scans were performed. Success of OVX was determined at necropsy by determination of uterine horn weight compared to SHAM rats. This study was approved by the Animal Care Committee of Brock University and was conducted in accordance with the Canadian Council on Animal Care.

### 2.2. Micro-Computed Tomography

The right hemi-mandible of 6-month old rats was hydrated in phosphate buffered saline (Sigma, St. Louis, MO, USA) at room temperature for two hours and subsequently wrapped in parafilm wax prior to ex vivo scanning. Micro-computed tomography of the hemi-mandible was performed using the μCT scanner (SkyScan 1176, Bruker microCT, Kontich, Belgium). High quality images of the trabecular bone were acquired with an isotropic voxel size of 9 μm, 80 kV, 298 μA, 1550 ms exposure time, 0.2° rotation step over 180°, and a 2 mm Al filter. The resulting images were reconstructed using NRecon software (v.1.6.9.10, Bruker microCT, Kontich, Belgium). A Gaussian filter was applied to the images with a smoothing kernel of 4, ring artifact reduction of 8%, beam hardening correction of 35%, defect pixel masking of 3%, and a dynamic image range from 0.000 to 0.044 (attenuation coefficient).

**Figure 1:**
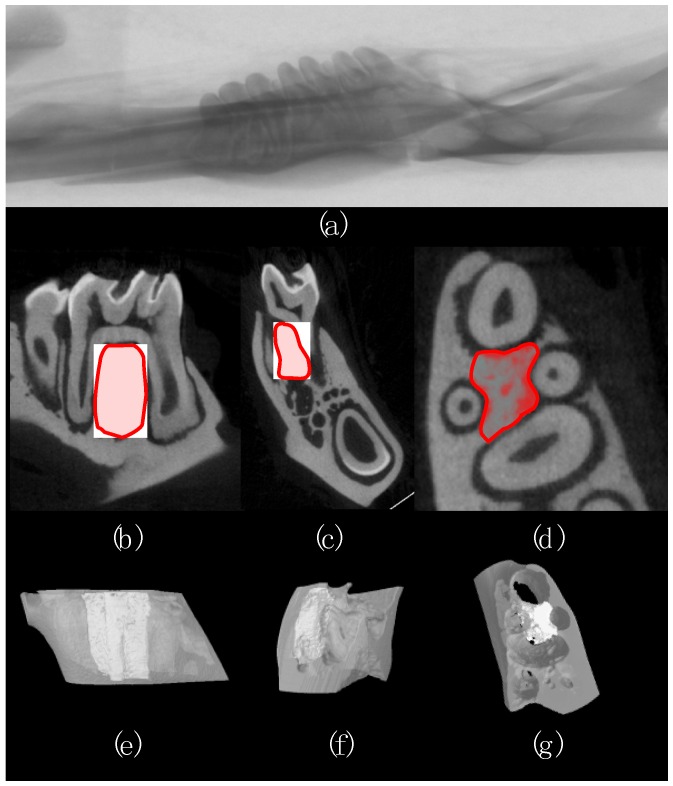
Representative image of a rat (**a**) hemi mandible; (**b**–**d**) sagittal, coronal, and transverse cross-section of the first molar with the region of interest (ROI) in the inter-radicular septum defined in red; (**e**–**g**) 3-dimensional representation of the volume (grey) surrounding the ROI (white).

The region of interest (ROI) to be analyzed began at the furcation roof at which the roots of the first molar became visibly independent structures. An interpolated hand-drawn shape was drawn to contour the root surfaces in contact with the alveolar bone in the inter-radicular septum. The ROI extended until the end of the mesial root (Figure 1). A global threshold value of 102 was set to binarize bone tissue from non-bone tissue. Trabecular microarchitectural measurements, including percent bone volume (BV/TV), connectivity density (Conn. D), degree of anisotropy (DA), trabecular number (Tb. N.), trabecular separation (Tb. Sp.), and trabecular thickness (Tb. Th.), were quantified using 3D analysis of the defined ROI and are summarized in Table 2 (CT Analyzer, v.1.14.4.1 + (64-bit), SkyScan, Bruker microCT, Kontich, Belgium). Results were reported as per the guidelines for assessment of bone microarchitecture of rodents using μCT [54].

### 2.3. Statistical Analyses

The effects of hormone status (with two levels: SHAM or OVX) and diet (with two levels: Flaxseed oil or Menhaden oil) and their interaction for each dependent variable were examined using a two-way ANOVA. A Bonferroni post-hoc test was used to test the main effects of hormone status and diet. All statistical analyses were performed using SPSS Statistics (v. 22, IBM). Significance was set at *p* < 0.05. All results are expressed as Mean ± SD.

## 3. Results

**Figure 2:**
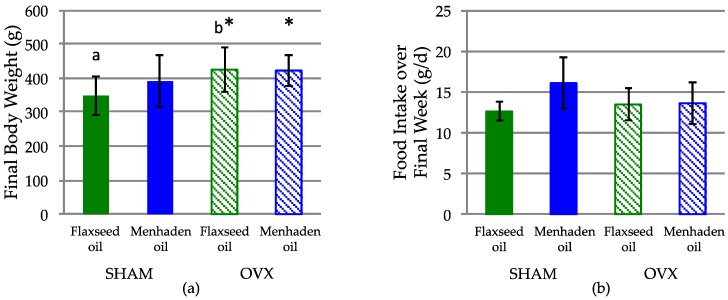
Final (**a**) body weight and (**b**) food intake of 6-month old SHAM and OVX rats randomized to diets with flaxseed oil or menhaden oil. A significant main effect of hormone status was observed for final body weight (* denotes a difference between both SHAM groups versus both OVX groups, *p* < 0.05). Within rats receiving a diet with flaxseed oil, rats randomized to OVX (424.67 ± 65.22 g) had significantly greater final body weight compared to SHAM (348.92 ± 57.51 g) (a versus b denotes a difference between these two groups fed flaxseed oil, *p* < 0.05). Within rats receiving a diet with menhaden oil, the body weight of rats randomized to OVX (422.17 ± 47.05 g) was not significantly different compared to SHAM (390.83 ± 75.82 g). No differences were observed among groups for food intake over the final week of the study period. All data are expressed as Mean ± SD.

No significant interaction of diet and hormone status was observed for final body weight, food intake, uterine weight or for any of the trabecular microarchitecture outcomes of the mandible. As expected, there was a significant main effect of hormone status on final body weight, whereby OVX rats (423.42 ± 56.14 g) weighed significantly more than SHAM rats (369.88 ± 66.67 g) (*p* = 0.005) (Figure 2). Within rats receiving a diet with flaxseed oil, rats randomized to OVX (424.67 ± 65.22 g) had significantly greater final body weight compared to SHAM (348.92 ± 57.51 g). Within rats receiving a diet with menhaden oil, the body weight of rats randomized to OVX (422.17 ± 47.05 g) was not significantly different compared to SHAM (390.83 ± 75.82 g). OVX rats had significantly lower uterine weight at necropsy (104 ± 93 mg) compared to SHAM rats (650 ± 150 mg), confirming successful OVX at three months of age. Food intake over the final week of the study was not affected by either diet or hormone status (Figure 2).

As shown in Table 3, diet had no significant main effect on any of the trabecular microarchitecture outcomes of the mandible. As expected, OVX had a significant main effect on several outcomes of the mandible compared to SHAM group and post hoc analysis revealed that only OVX rats fed menhaden oil were significantly different from SHAM rats fed menhaden oil. Specifically, OVX rats fed menhaden oil had greater Tb. Th. (*p* < 0.001), and Tb. Sp. (*p* < 0.05), and reduced BV/TV (*p* < 0.05), Conn. D. (*p* < 0.001), and Tb. N. (*p* < 0.05) compared to SHAM rats fed menhaden oil.

## 4. Discussion

Our findings showed that there was no interaction of dietary oil source and hormone status for any of the outcomes assessed, including food intake, final body weight and trabecular microarchitecture outcomes. Consistent with the literature, the hormone status of rats had a significant effect on all trabecular microarchitecture outcomes of the alveolar bone of the inter-radicular septum under the first molar [47,48,49,50,51,52,53]. However, the differences in microarchitecture outcomes between SHAM and OVX rats existed only in those fed menhaden oil. This suggests that when *n*-3 PUFA is supplied in the diet as ALA from flaxseed oil, and not EPA + DHA from menhaden oil, the diet may protect against OVX-induced bone loss at the mandible.

Several reviews have summarized that *n*-3 PUFA and their lipid mediators play important roles in the regulation of bone metabolism in growing and in OVX rats [55]. Potential mechanisms linking *n*-3 PUFA intake and bone metabolism include altered fatty acid composition of bone cell membranes, modified activity and differentiation of the osteoblast and osteoclast, and anti-inflammatory eicosanoid production [55]. Generally, in studies of growing rats or of OVX rats, a benefit to the long bones of the skeleton and/or the lumbar spine is observed with supplementation of either purified DHA [56,57,58] or fish oils with a high DHA content [59,60] and a detriment is found with purified EPA supplementation [56,61] or with fish oils with a high EPA content [60], although the mechanisms behind these different effects is unclear. Feeding young, growing rats flaxseed oil for eight weeks improves trabecular microarchitecture of the tibia compared to control fed rats, but these improvements are not significantly different from changes observed in rats fed menhaden oil [60]. These results suggest that bone is responsive to *n*-3 supplementation during periods of rapid growth, regardless of the oil source [60]. The current experiment expands on this concept and is the first to study bone microarchitecture in response to oils with different types of *n*-3 fatty acids when the intervention occurs throughout periods of rapid growth and following hormone-induced bone loss.

As expected, OVX caused a reduction in BV/TV, Tb. N., and Conn. D. and an increase in Tb. Sp. compared to SHAM rats. Unexpectedly, OVX caused a significant increase in Tb. Th. of the alveolar bone. This finding is not consistent with the literature. Previous studies have shown either decreases [47,48,49,50,52,53] or no significant difference [51,62] in Tb. Th. compared to SHAM rats, although no study has investigated the longitudinal change in Tb. Th. of the alveolar bone in the same rat using μCT. In most studies reporting decreases or no change in Tb. Th., rats were older (4–7 months of age) when randomized to SHAM or OVX surgery [47,48,50,51,52,62] and so the increases in Tb. Th. observed in this study may be due to the timing of OVX. Although the time points in our study are similar to those commonly used in OVX rat studies of PUFA interventions and long bone health [63,64,65], OVX at three months of age causes a premature switch to bone remodeling from normal growth [66]. Therefore, for future study of the alveolar bone, OVX after three months of age may be necessary to limit the disruption to normal trabecular growth and development that may still be occurring at the mandible.

Strengths of this study involve the long time course of supplementation and the design of the intervention diets. Rats were fed the experimental diets from a young age, coinciding with periods of rapid bone growth, through randomization to SHAM or OVX surgery, and for a three-month period following surgery. This was done to more closely mimic human dietary patterns, which may not change over time [67]. Moreover, the diets were designed to ensure that the macronutrient profile, micronutrient profile, total SFA, MUFA, and PUFA were matched. The *n*-6 to *n*-3 PUFA ratio of each intervention diet was also matched at 5 to 1 to mimic a diet that can be consumed through a healthful dietary pattern. A further strength of this study was the use of μCT at a spatial resolution of 9 μm to quantify microarchitecture outcomes of the mandibular alveolar bone. The use of such high resolutions for the acquisition of images limits partial volume effects and other artifacts of μCT scanning that can blur the definition between bone and non-bone tissue within each individual voxel, ultimately over or underestimating the 3D trabecular outcomes. A limitation of this study was that we cannot elucidate the effects of specific fatty acids on the structure of the alveolar bone in rats. Further study is needed to identify the role of specific fatty acids or combinations of fatty acids involved with these effects.

## 5. Conclusions

In summary, feeding rats flaxseed oil from one to six months of age provides protection against the OVX-induced bone loss at the mandible when OVX is performed at three months of age. Similar protection to the trabecular bone microarchitecture is not observed when rats are fed menhaden oil. These results suggest a benefit to incorporating plant sources of *n*-3 PUFA in the diet during the lifespan to help support the structural integrity of the mandible, and thus promoting retention of teeth.

## Figures and Tables

**Table 1:** Components of intervention diets.

Component (g/kg)	Flaxseed Oil	Menhaden Oil
Casein + l-Cysteine	203	203
Corn Starch	357	357
Maltodextrin	132	132
Sucrose	100	100
Cellulose	50	50
Mineral Mix ^1^	35	35
Vitamin Mix ^2^	10	10
Choline Bitartrate	2.5	2.5
TBHQ	0.022	0.022
Safflower Oil	45	70
Cottonseed Oil	43	4
Flaxseed Oil	22	-
Menhaden Oil	-	36
SFA	18.25	17.47
MUFA	18.16	18.07
PUFA	73.55	72.42
*n*-6	61.32	58.21
*n*-3	12.23	11.71
*n*-6:*n*-3	5.01	4.97

^1^ AIN-93G-MX (94046); ^2^ AIN-93-VX (94047).

**Table 2:** Summary of trabecular microarchitecture outcomes measured by μCT.

Microarchitecture Measurement	Unit	Explanation
Percent bone volume (BV/TV)	%	Percentage of area within ROI occupied by bone
		*Greater BV/TV is considered positive for bone health*
Connectivity density (Conn. D)	mm^−3^	Number of redundant connections between trabecular structures per unit volume
		*Greater Conn. D is considered positive for bone health*
Degree of anisotropy (DA)	No unit	Orientation of the trabecular network, 1-isotropic (lacking orientation), >1 = anisotropic (highly orientated)
		*Greater DA is considered positive for bone health*
Trabecular number (Tb. N.)	mm^−1^	Average number of trabeculae per unit length
		*Greater Tb. N. is considered positive for bone health*
Trabecular separation (Tb. Sp.)	mm	Average distance between trabeculae
		*Greater Tb. Sp. is considered negative for bone health*
Trabecular thickness (Tb. Th.)	mm	Average thickness of trabeculae
		*Greater Tb. Th. is considered positive for bone health*

**Table 3:** Trabecular microarchitecture outcomes of the inter-radicular cavity below the first molar of the mandible in SHAM and OVX rats.

Trabecular Outcome	SHAM		OVX		*p* Value		
	Flaxseed Oil	Menhaden Oil	Flaxseed Oil	Menhaden Oil	Interaction	Hormone Status	Diet
**BV/TV (%)**	55.87 (5.83)	59.09 (4.82)	53.35 (7.61)	52.22 (5.29) *	NS	0.05	NS
**Conn. D (mm^−3^)**	915.66 (445.40)	1420.86 (858.96)	545.43 (446.03)	541.75 (230.40) **	NS	0.001	NS
**DA**	1.32 (0.06)	1.33 (0.06)	1.38 (0.08)	1.33 (0.06)	NS	NS	NS
**Tb. N. (mm^−1^)**	11.709 (1.523)	12.847 (1.463)	11.011 (1.923)	10.764 (1.363) *	NS	0.05	NS
**Tb. Sp. (mm)**	0.173 (0.056)	0.137 (0.056)	0.203 (0.082)	0.221 (0.083) *	NS	0.05	NS
**Tb. Th. (mm)**	0.048 (0.001)	0.046 (0.002)	0.049 (0.002)	0.049 (0.001) **	NS	0.001	NS

No significant (NS) interaction or main effect of diet was observed for any of the trabecular microarchitecture outcomes measured. Hormone status had a significant main effect. Rats randomized to OVX had a significantly lower BV/TV, Conn. D, and Tb. N., and significantly greater Tb. Sp. and Tb. Th. compared to SHAM. All data are expressed as Mean (SD). Significantly different from SHAM rats receiving the same diet (*, *p* < 0.05; **, *p* < 0.001).

## References

[1] Darcey J., Horner K., Walsh T., Southern H., Marjanovic E.J., Devlin H. (2013). Tooth loss and osteoporosis: To assess the association between osteoporosis status and tooth number. Br. Dent. J..

[2] Drozdzowska B., Pluskiewicz W., Michno M. (2006). Tooth count in elderly women in relation to their skeletal status. Maturitas.

[3] Gur A., Nas K., Kayhan O., Atay M.B., Akyuz G., Sindal D., Aksit R., Oncel S., Dilsen G., Cevik R. (2003). The relation between tooth loss and bone mass in postmenopausal osteoporotic women in Turkey: A multicenter study. J. Bone Miner. Metab..

[4] Henriques P.S., Pinto Neto A.M. (2011). Association between tooth loss and bone mineral density in Brazilian postmenopausal women. J. Clin. Med. Res..

[5] Inagaki K., Kurosu Y., Kamiya T., Kondo F., Yoshinari N., Noguchi T., Krall E.A., Garcia R.I. (2001). Low metacarpal bone density, tooth loss, and periodontal disease in Japanese women. J. Dent. Res..

[6] Iwasaki M., Nakamura K., Yoshihara A., Miyazaki H. (2012). Change in bone mineral density and tooth loss in japanese community-dwelling postmenopausal women: A 5-year cohort study. J. Bone Miner. Metab..

[7] Jang K.M., Cho K.H., Lee S.H., Han S.B., Han K.D., Kim Y.H. (2015). Tooth loss and bone mineral density in postmenopausal south korean women: The 2008–2010 korea national health and nutrition examination survey. Maturitas.

[8] Krall E.A., Dawson-Hughes B., Papas A., Garcia R.I. (1994). Tooth loss and skeletal bone density in healthy postmenopausal women. Osteoporos. Int..

[9] Krall E.A., Garcia R.I., Dawson-Hughes B. (1996). Increased risk of tooth loss is related to bone loss at the whole body, hip, and spine. Calcif. Tissue Int..

[10] Mohammad A.R., Hooper D.A., Vermilyea S.G., Mariotti A., Preshaw P.M. (2003). An investigation of the relationship between systemic bone density and clinical periodontal status in post-menopausal asian-american women. Int. Dent. J..

[11] Nicopoulou-Karayianni K., Tzoutzoukos P., Mitsea A., Karayiannis A., Tsiklakis K., Jacobs R., Lindh C., van der Stelt P., Allen P., Graham J. (2009). Tooth loss and osteoporosis: The osteodent study. J. Clin. Periodontol..

[12] Taguchi A., Suei Y., Ohtsuka M., Otani K., Tanimoto K., Hollender L.G. (1999). Relationship between bone mineral density and tooth loss in elderly Japanese women. Dentomaxillofacial Radiol..

[13] Brennan D.S., Singh K.A., Liu P., Spencer A. (2010). Fruit and vegetable consumption among older adults by tooth loss and socio-economic status. Aust. Dent. J..

[14] Sahyoun N.R., Lin C.L., Krall E. (2003). Nutritional status of the older adult is associated with dentition status. J. Am. Diet. Assoc..

[15] Sheiham A., Steele J. (2001). Does the condition of the mouth and teeth affect the ability to eat certain foods, nutrient and dietary intake and nutritional status amongst older people?. Public Health Nutr..

[16] Yoshihara A., Watanabe R., Nishimuta M., Hanada N., Miyazaki H. (2005). The relationship between dietary intake and the number of teeth in elderly Japanese subjects. Gerodontology.

[17] Zhu Y., Hollis J.H. (2014). Tooth loss and its association with dietary intake and diet quality in american adults. J. Dent..

[18] Johnston B.D., Ward W.E. (2015). The ovariectomized rat as a model for studying alveolar bone loss in postmenopausal women. BioMed Res. Int..

[19] Liu S., Manson J.E., Lee I.M., Cole S.R., Hennekens C.H., Willett W.C., Buring J.E. (2000). Fruit and vegetable intake and risk of cardiovascular disease: The women’s health study. Am. J. Clin. Nutr..

[20] Genkinger J.M., Platz E.A., Hoffman S.C., Comstock G.W., Helzlsouer K.J. (2004). Fruit, vegetable, and antioxidant intake and all-cause, cancer, and cardiovascular disease mortality in a community-dwelling population in washington county, maryland. Am. J. Epidemiol..

[21] Benetou V., Orfanos P., Feskanich D., Michaelsson K., Pettersson-Kymmer U., Eriksson S., Grodstein F., Wolk A., Bellavia A., Ahmed L.A.I. (2016). Fruit and vegetable intake and hip fracture incidence in older men and women: The CHANCES project. J. Bone Miner. Res..

[22] Virtanen J.K., Mozaffarian D., Willett W.C., Feskanich D. (2012). Dietary intake of polyunsaturated fatty acids and risk of hip fracture in men and women. Osteoporos. Int..

[23] Orchard T.S., Cauley J.A., Frank G.C., Neuhouser M.L., Robinson J.G., Snetselaar L., Tylavsky F., Wactawski-Wende J., Young A.M., Lu B. (2010). Fatty acid consumption and risk of fracture in the women’s health initiative. Am. J. Clin. Nutr..

[24] Jarvinen R., Tuppurainen M., Erkkila A.T., Penttinen P., Karkkainen M., Salovaara K., Jurvelin J.S., Kroger H. (2012). Associations of dietary polyunsaturated fatty acids with bone mineral density in elderly women. Eur. J. Clin. Nutr..

[25] Mangano K., Kerstetter J., Kenny A., Insogna K., Walsh S.J. (2014). An investigation of the association between omega 3 FA and bone mineral density among older adults: Results from the national health and nutrition examination survey years 2005–2008. Osteoporos. Int..

[26] Rousseau J.H., Kleppinger A., Kenny A.M. (2009). Self-reported dietary intake of omega-3 fatty acids and association with bone and lower extremity function. J. Am. Geriatr. Soc..

[27] Nawata K., Yamauchi M., Takaoka S., Yamaguchi T., Sugimoto T. (2013). Association of polyunsaturated fatty acid intake with bone mineral density in postmenopausal women. Calcif. Tissue Int..

[28] Longo A.B., Ward W.E. (2016). PUFAs, bone mineral density, and fragility fracture: Findings from human studies. Adv. Nutr..

[29] Institute of Medicine (2005). Dietary Reference Intakes for Energy, Carbohydrate, Fiber, Fat, Fatty Acids, Cholesterol, Protein, and Amino Acids (Macronutrients).

[30] Dodington D.W., Fritz P.C., Sullivan P.J., Ward W.E. (2015). Higher intakes of fruits and vegetables, beta-carotene, vitamin C, alpha-tocopherol, EPA, and DHA are positively associated with periodontal healing after nonsurgical periodontal therapy in nonsmokers but not in smokers. J. Nutr..

[31] Hamazaki K., Itomura M., Sawazaki S., Hamazaki T. (2006). Fish oil reduces tooth loss mainly through its anti-inflammatory effects?. Med. Hypotheses.

[32] Iwasaki M., Taylor G.W., Moynihan P., Yoshihara A., Muramatsu K., Watanabe R., Miyazaki H. (2011). Dietary ratio of *n*-6 to polyunsaturated fatty acids and periodontal disease in community-based older Japanese: A 3-year follow-up study. Prostaglandins Leukot. Essent. Fat. Acids.

[33] Iwasaki M., Yoshihara A., Moynihan P., Watanabe R., Taylor G.W., Miyazaki H. (2010). Longitudinal relationship between dietary omega-3 fatty acids and periodontal disease. Nutrition.

[34] Naqvi A.Z., Buettner C., Phillips R.S., Davis R.B., Mukamal K.J. (2010). *n*-3 fatty acids and periodontitis in us adults. J. Am. Diet. Assoc..

[35] Rosenstein E.D., Kushner L.J., Kramer N., Kazandjian G. (2003). Pilot study of dietary fatty acid supplementation in the treatment of adult periodontitis. Prostaglandins Leukot. Essent. Fat. Acids.

[36] Campan P., Planchand P.O., Duran D. (1997). Pilot study on polyunsaturated fatty acids in the treatment of human experimental gingivitis. J. Clin. Periodontol..

[37] Lucas E.A., Wild R.D., Hammond L.J., Khalil D.A., Juma S., Daggy B.P., Stoecker B.J., Arjmandi B.H. (2002). Flaxseed improves lipid profile without altering biomarkers of bone metabolism in postmenopausal women. J. Clin. Endocrinol. Metab..

[38] Dodin S., Lemay A., Jacques H., Legare F., Forest J.C., Masse B. (2005). The effects of flaxseed dietary supplement on lipid profile, bone mineral density, and symptoms in menopausal women: A randomized, double-blind, wheat germ placebo-controlled clinical trial. J. Clin. Endocrinol. Metab..

[39] Brooks J.D., Ward W.E., Lewis J.E., Hilditch J., Nickell L., Wong E., Thompson L.U. (2004). Supplementation with flaxseed alters estrogen metabolism in postmenopausal women to a greater extent than does supplementation with an equal amount of soy. Am. J. Clin. Nutr..

[40] Vanpapendorp D.H., Coetzer H., Kruger M.C. (1995). Biochemical profile of osteoporotic patients on essential fatty-acid supplementation. Nutr. Res..

[41] Kruger M.C., Coetzer H., de Winter R., Gericke G., van Papendorp D.H. (1998). Calcium, gamma-linolenic acid and eicosapentaenoic acid supplementation in senile osteoporosis. Aging (Milano).

[42] Appleton K.M., Fraser W.D., Rogers P.J., Ness A.R., Tobias J.H. (2011). Supplementation with a low-moderate dose of long-chain pufa has no short-term effect on bone resorption in human adults. Br. J. Nutr..

[43] Bassey E.J., Littlewood J.J., Rothwell M.C., Pye D.W. (2000). Lack of effect of supplementation with essential fatty acids on bone mineral density in healthy pre- and postmenopausal women: Two randomized controlled trials of efacal v. Calcium alone. Br. J. Nutr..

[44] Kalu D.N. (1991). The ovariectomized rat model of postmenopausal bone loss. Bone Miner..

[45] Thompson D.D., Simmons H.A., Pirie C.M., Ke H.Z. (1995). FDA guidelines and animal models for osteoporosis. Bone.

[46] Dodd D.Z., Rowe D.J. (2013). The relationship between postmenopausal osteoporosis and periodontal disease. J. Dent. Hyg..

[47] Irie K., Sakakura Y., Tsuruga E., Hosokawa Y., Yajima T. (2004). Three-dimensional changes of the mandible and alveolar bone in the ovariectomized rat examined by micro-focus computed tomography. J. Jpn. Soc. Periodontol..

[48] Liu X.L., Li C.L., Lu W.W., Cai W.X., Zheng L.W. (2015). Skeletal site-specific response to ovariectomy in a rat model: Change in bone density and microarchitecture. Clin. Oral Implant. Res..

[49] Liu Z., Yan C., Kang C., Zhang B., Li Y. (2015). Distributional variations in trabecular architecture of the mandibular bone: An in vivo micro-ct analysis in rats. PLoS ONE.

[50] Mavropoulos A., Kiliaridis S., Rizzoli R., Ammann P. (2014). Normal masticatory function partially protects the rat mandibular bone from estrogen-deficiency induced osteoporosis. J. Biomech..

[51] Mavropoulos A., Rizzoli R., Ammann P. (2007). Different responsiveness of alveolar and tibial bone to bone loss stimuli. J. Bone Miner. Res..

[52] Tanaka M., Toyooka E., Kohno S., Ozawa H., Ejiri S. (2003). Long-term changes in trabecular structure of aged rat alveolar bone after ovariectomy. Oral Surg. Oral Med. Oral Pathol. Oral Radiol. Endod..

[53] Yang J., Pham S.M., Crabbe D.L. (2003). Effects of oestrogen deficiency on rat mandibular and tibial microarchitecture. Dentomaxillofacial Radiol..

[54] Bouxsein M.L., Boyd S.K., Christiansen B.A., Guldberg R.E., Jepsen K.J., Muller R. (2010). Guidelines for assessment of bone microstructure in rodents using micro-computed tomography. J. Bone Miner. Res..

[55] Lau B.Y., Cohen D.J., Ward W.E., Ma D.W. (2013). Investigating the role of polyunsaturated fatty acids in bone development using animal models. Molecules.

[56] Yamada Y., Fushimi H., Inoue T., Matsuyama Y., Kameyama M., Minami T., Okazaki Y., Noguchi Y., Kasama T. (1995). Effect of eicosapentaenoic acid and docosahexaenoic acid in diabetic osteopenia. Diabetes Res. Clin. Pract..

[57] Li Y., Seifert M.F., Lim S.Y., Salem N., Watkins B.A. (2010). Bone mineral content is positively correlated to *n*-3 fatty acids in the femur of growing rats. Br. J. Nutr..

[58] Poulsen R.C., Firth E.C., Rogers C.W., Moughan P.J., Kruger M.C. (2007). Specific effects of gamma-linolenic, eicosapentaenoic, and docosahexaenoic ethyl esters on bone post-ovariectomy in rats. Calcif. Tissue Int..

[59] Kruger M.C., Schollum L.M. (2005). Is docosahexaenoic acid more effective than eicosapentaenoic acid for increasing calcium bioavailability?. Prostaglandins Leukot. Essent. Fat. Acids.

[60] Lukas R., Gigliotti J.C., Smith B.J., Altman S., Tou J.C. (2011). Consumption of different sources of omega-3 polyunsaturated fatty acids by growing female rats affects long bone mass and microarchitecture. Bone.

[61] Poulsen R.C., Kruger M.C. (2006). Detrimental effect of eicosapentaenoic acid supplementation on bone following ovariectomy in rats. Prostaglandins Leukot. Essent. Fat. Acids.

[62] Tanaka M., Ejiri S., Toyooka E., Kohno S., Ozawa H. (2002). Effects of ovariectomy on trabecular structures of rat alveolar bone. J. Periodontal Res..

[63] Watkins B.A., Li Y., Seifert M.F. (2006). Dietary ratio of *n*-6/*n*-3 PUFAs and docosahexaenoic acid: Actions on bone mineral and serum biomarkers in ovariectomized rats. J. Nutr. Biochem..

[64] Sacco S.M., Jiang J.M., Reza-Lopez S., Ma D.W., Thompson L.U., Ward W.E. (2009). Flaxseed combined with low-dose estrogen therapy preserves bone tissue in ovariectomized rats. Menopause.

[65] Sacco S.M., Jiang J.M., Thompson L.U., Ward W.E. (2012). Flaxseed does not enhance the estrogenic effect of low-dose estrogen therapy on markers of uterine health in ovariectomized rats. J. Med. Food.

[66] Longo A.B., Sacco S.M., Salmon P.L., Ward W.E. (2016). Longitudinal use of micro-computed tomography does not alter microarchitecture of the proximal tibia in sham or ovariectomized sprague-dawley rats. Calcif. Tissue Int..

[67] Mikkila V., Rasanen L., Raitakari O.T., Pietinen P., Viikari J. (2005). Consistent dietary patterns identified from childhood to adulthood: The cardiovascular risk in young finns study. Br. J. Nutr..

